# Artificial Intelligence for Lentigo Maligna: Automated Margin Assessment via Sox-10-Based Melanocyte Density Mapping

**DOI:** 10.3390/dermatopathology13010001

**Published:** 2025-12-19

**Authors:** Rieke Löper, Lennart Abels, Daniel Otero Baguer, Felix Bremmer, Michael P. Schön, Christina Mitteldorf

**Affiliations:** 1 Department of Dermatology, Venereology and Allergology, University Medical Center Göttingen, Robert-Koch-Straße 40, 37075 Göttingen, Germany; rieke.loeper@stud.uni-goettingen.de (R.L.);; 2 Center for Industrial Mathematics, University Bremen, Bibliothekstraße 5, 28359 Bremen, Germany; 3 Department of Pathology, University Medical Center Göttingen, Robert-Koch-Straße 40, 37075 Göttingen, Germany

**Keywords:** lentigo maligna, melanocyte density, artificial intelligence, deep learning, margin assessment

## Abstract

Lentigo maligna (LM) is a melanoma in situ with high cumulative sun damage. Histological evaluation of resection margins is difficult and time-consuming. Melanocyte density (MD) is a suitable, quantifiable, and reproducible diagnostic criterion. In this retrospective single-centre study, we investigated whether an artificial intelligence (AI) tool can support the assessment of LM. Training and evaluation were based on MD in Sox-10-stained digitalised slides. In total, 86 whole slide images (WSIs) from LM patients were annotated and used as a training set. The test set consisted of 177 slides. The tool was trained to detect the epidermis, measure its length, and determine the MD. A cut-off of ≥30 melanocytes per 0.5 mm of epidermis length was defined as positive. Our AI model automatically recognises the epidermis and measures the MD. The model was trained on nuclear immunohistochemical signals and can also be applied to other nuclear stains, such as PRAME or MITF. The WSI is automatically visualised by a three-colour heat map with a subdivision into low, borderline, and high melanocyte density. The cut-offs can be adjusted individually. Compared to manually counted ground truth MD, the AI model achieved high sensitivity (87.84%), specificity (72.82%), and accuracy (79.10%), and an area under the curve (AUC) of 0.818 in the test set. This automated tool can assist (dermato) pathologists by providing a quick overview of the WSI at first glance and making the time-consuming assessment of resection margins more efficient and more reproducible. The AI model can provide significant benefits in the daily routine workflow.

## Simple Summary

Since lentigo maligna, a melanoma in situ, occurs primarily on sun-damaged skin, it is often difficult to distinguish it histologically from the latter, especially in peripheral areas, as both have similar histological characteristics. Artificial intelligence is already widely used in dermatopathology. This study, therefore, examines whether such a tool can be helpful in assessing the margins of lentigo maligna. Our tool is primarily based on the detection of epidermal melanocytes in digitised slides, as increased melanocyte density is a decisive criterion for malignancy in lentigo maligna. It reliably detects these and also provides colour coding of the melanocytes in a traffic light system, with areas of low, borderline, and increased density. This procedure should be improved in further studies, but it has the potential to make daily workflows more time-efficient and to provide support in cases of doubt.

## 1. Introduction

Lentigo maligna (LM) belongs to the group of high cumulative sun damage (h-CSD) melanoma and occurs typically in older patients [1,2]. The standard treatment of LM is a surgical excision with the intention of removing the tumour completely to reduce the risk of recurrence and progression [3,4]. However, as lesions are often large and occur in visible areas, extensive excision is undesirable due to aesthetic concerns.

Modern histopathology routinely includes immunohistochemical stains, such as Sox-10 (Sry-related HMg box gene 10), Melan-A (Melanoma antigen recognised by T cells 1), and PRAME (Preferentially Expressed Antigen in Melanoma) [5]. In addition to the diagnosis and exclusion of an invasive component, particular attention is paid to the assessment of tumour margins. However, LM assessment is challenging and time-consuming. An interobserver study reported only moderate concordance for margin analysis [6]. Even though LM generally shows a higher number of atypical melanocytes compared to sun-damaged skin, a clear cut-off value for melanocyte density (MD) has not yet been established (reviewed by Löper et al.) [7]. Contributing factors are heterogeneous study designs and different staining techniques. As the surrounding skin usually also shows melanocyte hyperplasia due to sun exposure, the more subtle features of LM in the margins are often difficult to recognise. This may be one of the reasons for the high interobserver variability [8,9,10,11]. Especially in large tumours and re-excision specimens, tissue is often cut with separate margins, which complicates interpretation because these areas cannot be directly compared with the tumour centre.

It appears that artificial intelligence (AI) may become increasingly important for the diagnosis of cutaneous tumours [12,13,14]. Numerous publications suggest that AI-assisted pathology will become an integral part of future dermatopathological evaluations [15,16,17]. Previous publications have already shown that AI can diagnose at or above the level of dermatopathologists [18,19]. In the past, non-melanocytic entities such as basal cell carcinoma, dermal nevi, and seborrheic keratosis have already been successfully evaluated with AI [20,21,22,23,24]. However, it has also been applied to melanocytic lesions in conjunction with dermatoscopic images, reflectance confocal microscopy, and histologic evaluations [25,26,27,28,29,30,31,32,33,34,35,36,37,38,39,40,41]. It has also been shown in the histopathological examination of melanoma that a convolutional neural network has achieved good results in diagnosis [18]. A review of selected papers on AI and melanoma concluded that while dermatopathologists cannot be replaced, AI support will play an important role and will be a tool in the assessment of melanocytic lesions in the future [25]. Most studies on histologic assessment of cutaneous melanoma using deep learning models primarily use H&E staining as a reference [16].

To our knowledge, only one paper specifically refers to deep learning support in the evaluation of LM based on MD [42]. The authors concluded that AI could aid in LM pre-screening, although further development is needed.

The aim of this study was to evaluate whether an AI model can significantly improve the assessment of LM, especially resection margins, during routine histopathological examination. Specifically, the model was designed to identify the epidermis, to generate a melanocytic density (MD) on Sox-10-stained slides, and display it in a customisable, colour-coded heat map.

## 2. Materials and Methods

### 2.1. Training Set

**Figure 1:**
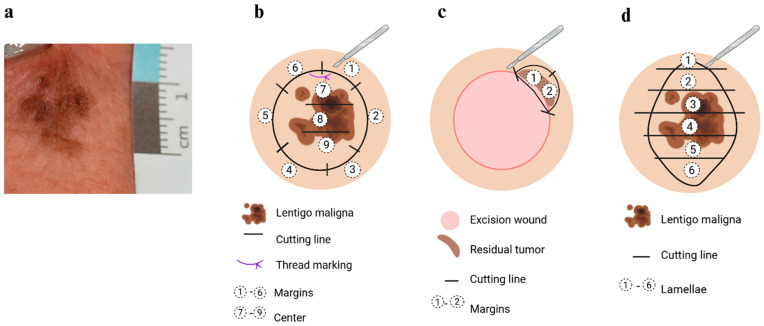
(**a**) Clinical appearance of lentigo maligna; (**b**) typical cutting and assessment of the centre and the margins of a large primary tumour; (**c**) re-excision in case of residual malignancy. In this example, an R0 status could not be achieved in the 12–1 o’clock and 1–2 o’clock ranges; (**d**) typical cutting and assessment of small spindle excision biopsies, which are processed in lamellae. Created in BioRender. Mitteldorf, C. (2025) https://BioRender.com/prv9047.

Selection of the training set:

A total of 86 slides from 18 patients diagnosed with LM between 2018 and 2021 at the Dermatopathology unit, Department of Dermatology, University Medical Centre Göttingen, were selected. We considered Sox-10-stained material from each patient, including the sample biopsies, the primary surgery, and the re-excisions, if performed. Thus, the available slides of the cases included both positive and negative cases. Most tumours were extensive and required inpatient excision at a university hospital (Figure 1a). Due to the generally large size of the specimens, most specimens were processed with centrally located sections (Figure 1b) and separately embedded margins (Figure 1b), and re-excisions were often necessary (Figure 1c). Small spindle-shaped specimens were processed in lamellae (Figure 1d).

Ground truth data:

The results from the original reports were extracted and used as ground truth data (GT-OR). Additionally, melanocyte density (MD) was counted for each slide in a blinded fashion. For this, the most densely affected area in the Sox-10-stained slide was selected, and the melanocytes over 0.50 mm were manually counted. Melanocytes appearing in nests, defined as such when there are three or more confluent melanocytes, were also counted. The presence of nests was also noted.

Slide annotation and AI training:

Digitalised Sox-10-stained slides were annotated and processed by colleagues at the Centre for Industrial Mathematics, Bremen, Germany, who were previously trained by an experienced dermatopathologist. The used AI is a residual network (ResNet34) with a deep learning approach. Sox-10 was chosen because of its clear nuclear signal, allowing future adaptation to other nuclear stains (e.g., PRAME and MITF) and its higher sensitivity and specificity for margins assessment in LM in comparison to MelanA [43]. For that, Sox-10-positive melanocytes were annotated, and the AI was trained to detect these signals over a defined distance. The model was also trained to identify the epidermis, since Sox-10 is not restricted to melanocytes, and only epidermal signals were relevant for this question.

During the training period, characteristics relating to the recognition of the epidermis and the detection of melanocytes had to be taken into account. Problems included false positive detection of contamination by colour markings, and poor epidermis recognition when sections were oblique, and the epidermis was cut tangentially. Furthermore, additional diagnoses (e.g., lentigo solaris, seborrheic keratosis, and (pigmented) actinic keratosis) were presented on the slides and had to also be considered in the training.

**Figure 2:**
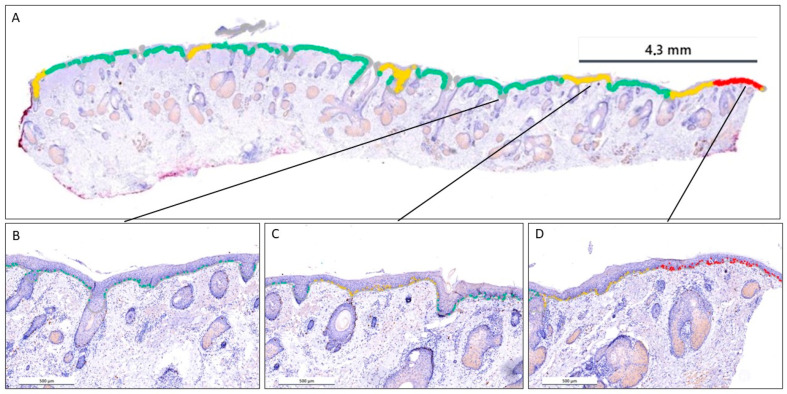
(**A**): The AI-generated colour-coded heat map illustrates the three categories of MD in a Sox-10-stained marginal section (×2.5). (**B**): green AI signal—low MD: <25 melanocytes/0.5 mm of epidermis (Sox-10; ×40). (**C**): yellow AI signal—borderline MD: 25–29 melanocytes/0.5 mm of epidermis (Sox-10; ×40). (**D**): red AI signal—high MD: ≥30 melanocytes/0.5 mm of epidermis (Sox-10; ×40).

**Figure 3:**
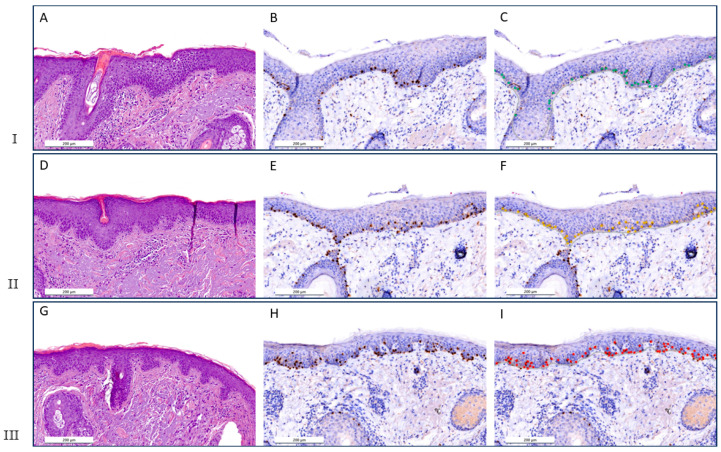
Detailed examples of the AI-generated colour-coded heat map for the three MD categories compared to H&E and Sox-10. **I**: Low MD (<25 melanocytes/0.5 mm of epidermis): (**A**) in H&E (×100), (**B**) in Sox-10 (×100), and (**C**) with AI annotation (Sox-10, ×100). **II**: Borderline MD (25–29 melanocytes/0.5 mm of epidermis): (**D**) in H&E (×100), (**E**) in Sox-10 (×100), and (**F**) with AI annotation (Sox-10, ×100). III: High MD (≥30 melanocytes/0.5 mm of epidermis): (**G**) in H&E (×100), (**H**) in Sox-10 (×100), and (**I**) with AI annotation (Sox-10, ×100).

False negative results occurred, especially when melanocytes in nests were not detected by the AI. The reason for this was that the signals were so close together that the AI could not distinguish them. Therefore, a second nest detection model was trained. The developed tool visualises MD in a colour-coded heat map (Figure 2). Detailed views of the three categories of the heat map are shown in Figure 3 in comparison to H&E and unmarked Sox-10 sections. An affected area is shown in red (≥30 melanocytes/0.5 mm of epidermis), a borderline finding in yellow (25–29 melanocytes/0.5 mm of epidermis), and a negative area is highlighted in green (<25 melanocytes/0.5 mm of epidermis). The reason why the cut-off values were set in this way is explained below. However, all cut-off values can be adjusted individually.

### 2.2. Architecture and Training

As described above, Sox-10-stained WSI were digitalised and annotated for epidermis and Sox-10-positive melanocytes. Based on these annotations, we trained two convolutional neural networks with a U-Net architecture for semantic segmentation, one for epidermis detection and one for melanocytic nuclei detection. The U-Net models followed the original encoder–decoder design with skip connections as proposed by Ronneberger et al. [44].

For both tasks, WSIs were tiled into patches of 512 × 512 pixels, but at different magnifications. The epidermis model was trained on image patches extracted at 10× magnification, whereas the nuclei model was trained on patches extracted at 20× magnification to better resolve nuclear detail. Patches were sampled preferentially from annotated areas to ensure a sufficient proportion of foreground pixels. During training, standard data augmentation (random rotations, flips, and mild variations in brightness and contrast) was applied to increase robustness to staining and scanning variability. Both models were optimised using the Adam optimiser with a learning rate of 10^−5^.

The epidermis model was trained to segment epidermis and adnexal epithelium versus background. The U-Net output is a probability map with values in [0, 1]. For inference, this probability map was thresholded at 0.5 to obtain a binary mask, where 0 corresponds to background and 1 to epidermis. From this binary mask, contours of the epidermis were extracted and used to define the regions in which melanocyte counts were evaluated.

The nuclei model was trained on manual annotations of Sox-10-positive melanocytic nuclei at 20× magnification. Again, a U-Net produced a per-pixel probability map, which was thresholded at 0.5 to obtain candidate nuclear regions. Because neighbouring nuclei often touch or overlap, an additional instance-separation step was applied. Local maxima in the smoothed probability map were first identified as putative nuclear centres. These centres were then used as seeds for a marker-controlled watershed on the inverted distance transform of the binary mask, which allowed separation of adjacent nuclei into individual instances. To restrict the analysis to junctional melanocytes, the previously obtained epidermal contour was used as a reference, and only nuclei whose centroid lay within 10 µm of the lower (basal) border of the epidermis were retained. All other nuclei were excluded from the MD computation.

### 2.3. Validation Strategy

For the development of the epidermis segmentation U-Net, the Sox-10 training dataset (2018–2021) was randomly split at the slide level into 80% training and 20% internal validation data. This internal validation subset was used to choose the model hyperparameters (number of epochs, degree of data augmentation, and threshold for binarisation of the probability map). After selection of the final hyperparameters, the epidermis model was retrained on the entire training dataset.

The nuclei segmentation U-Net was trained using the same 80%/20% random split of slides for initial hyperparameter selection. In a second step, the post-processing parameters for instance-separation (e.g., smoothing of the probability map, detection of local maxima, and watershed settings) were qualitatively fine-tuned by visual inspection on the full training dataset.

Due to the relatively low number of patients and slides, we were not able to define an additional independent validation cohort that was completely separate from the data used for model training and for the final evaluation. Consequently, the internal 80%/20% splits were used only to guide model development and fine-tuning, whereas the performance labelled as “training set” in the results section refers to the final algorithm applied to the complete training cohort.

### 2.4. Test Set

The test set consisted of 177 slides from 10 patients who underwent surgery for LM at the University Medical Centre Göttingen in the period between 2021 and 2022. The histopathological processing and Sox-10 staining protocols were identical to those of the training set. Ground truth data (GT-OR and GT-MD) and nest occurrence were collected in the same way as in the training set. The AI model generated the data for the test set.

### 2.5. Cut-Off Values for Melanocyte Density

In the literature, there is no defined cut-off for MD as a diagnostic criterion for LM for Sox-10 [7]. Only one paper mentioned a cut-off of 25 melanocytes per 0.5 mm for sections stained for microphthalmia-associated transcription factor (MITF) [45]. Others used H&E-stained sections to classify the likelihood of a melanoma in situ recurring according to MD [46]. They identified three risk groups: a high risk of recurrence at ≥30 melanocytes per 0.5 mm, an intermediate risk at 21–30 melanocytes per 0.5 mm, and a low risk at 0–20 melanocytes per 0.5 mm [46]. Although these values cannot be easily transferred to Sox-10, they can serve as a reference point. A further question of this study was, therefore, which cut-off value was most suitable for distinguishing LM from sun-damaged skin with Sox-10 staining. The developed tool allows a manual adjustment of the cut-offs and of tolerance ranges, providing flexibility based on staining variability or pathologist preference.

Finding a suitable cut-off:

The Youden Index can also be used to calculate the cut-off value that produces the most reliable results. It is defined as follows within the ROC:

Youden index = sensitivity + specificity − 1

The most accurate statement about an appropriate cut-off value is best made based on the sensitivity and specificity of the manual count in relation to the original findings. Applying the different sensitivities and specificities of the respective thresholds, the highest value and thus the best cut-off value was obtained from 30 melanocytes per 0.5 mm. This is consistent with values reported in the prior literature [46,47].

In order to have certainty in the assessment of the resection margins and to avoid overlooking borderline areas, an additional category was introduced in the AI model, which also provides an immediate representation of a slightly lower MD than the straight cut-off value of 30 melanocytes per 0.5 mm, namely 25–29 melanocytes per 0.5 mm. Anything below this value was considered a negative result.

## 3. Results

### 3.1. Training Set

Of the 86 slides, five (5.81%) were sample biopsies, 12 (13.95%) were obtained from the centre of the lesion, 66 (76.74%) were resection margins, and three (3.49%) were from spindle preparation. For detailed information, see the supplementary data (Table S1).

Comparison of original findings, AI-assisted melanocyte detection, and manual counts with each other:

To illustrate the comparison of GT-OR (ground truth data of the original findings), AI-MD (data generated by the AI), and GT-MD (manual cell count ground truth data) with each other using statistical variables, we chose sensitivity, specificity, and accuracy. For this purpose, the evaluation of two compared variables was labelled as true positive (TP), true negative (TN), false positive (FP), and false negative (FN). If GT-OR was included in the comparison, this is set as ground truth. When comparing AI-MD to GT-MD, the latter was set as ground truth. In the comparison, a deviation of ≥5 melanocytes was considered an inconsistent result.

The sensitivity indicates the proportion of true positive slides that are correctly identified as such, which can also be referred to as the true positive rate (TPR).

$$Sensitivity\;(TPR)=\frac{TP}{TP+FN}$$

Specificity is the proportion of true negative slides that are correctly identified as such. This is called the true negative rate (TNR).

$$Specificity\;(TNR)=\frac{TN}{TN+FP}$$

Accuracy is the proportion of correct statements compared to all original findings.

$$Accuracy=\frac{TP+TN}{TP+TN+FP+FN}$$

Accordingly, Table 1 summarises sensitivity, specificity, and accuracy based on this cut-off.

**Figure 4:**
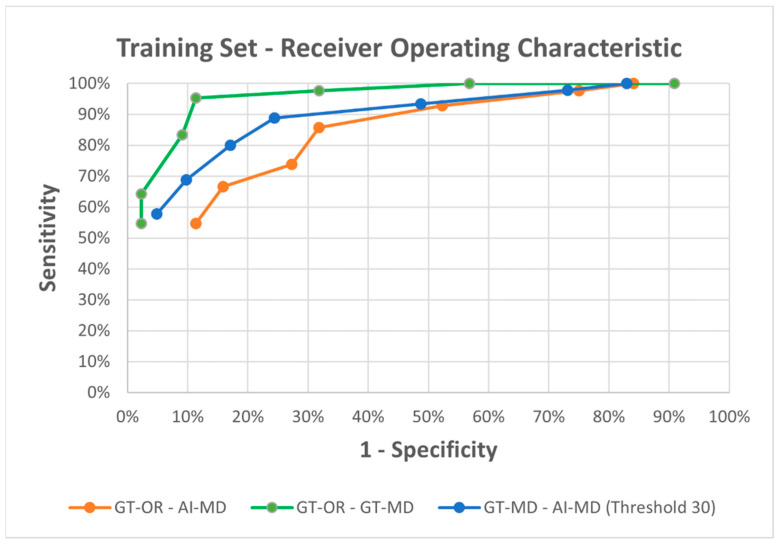
Receiver operator characteristic curve based on sensitivity (*y*-axis) and 1-specificity (*x*-axis) comparing AI-generated data based on melanocyte density, ground truth data, and manual cell count-based data in the training set. GT-OR = Ground truth original findings, AI-MD = Artificial intelligence detected melanocyte density; GT-MD = Ground truth melanocyte density.

Receiver operating characteristics:

We constructed ROC (receiver operating characteristic) curves for all possible combinations. The curves are shown in Figure 4.

The ROC curve is a graphical representation of the performance of a binary classification model for all thresholds, i.e., different cut-offs. For this, we need graphs with the sensitivity on the *y*-axis and 1—specificity on the *x*-axis. For each specific cut-off (ranging from 15 to 45 melanocytes per 0.5 mm, in increments of 5), we plot a point corresponding to sensitivity and 1—specificity. Sensitivity is the true positive rate, and 1—specificity is the false positive rate.

In the analysis of AI-MD versus GT-MD, the latter was considered the ground truth. However, since the results of GT-MD were also determined by the original data, a cut-off must first be set in this case, in which the results of the manual count were evaluated as ground truth. Since we found above that 30 melanocytes per 0.5 mm was the best cut-off, we set all data for comparison to the values obtained with a cut-off of 30 melanocytes per 0.5 mm.

An examination of the area under the curve (AUC) of the ROC provides insight into the good performance and diagnostic accuracy of the respective systems.

AUC (GT-OR vs. GT-MD) ≈ 0.856

AUC (GT-OR vs. AI-MD) ≈ 0.633

AUC (GT-MD (based on 30 melanocytes per 0.5 mm) vs. AI-MD) ≈ 0.699

The method of manual melanocyte counting in combination with the original findings therefore showed a large integral. This means that the single criterion of MD can already be used to establish a high level of agreement. A high correlation of the MD of ≥30 melanocytes per 0.5 mm with a positive result can be assumed. Looking at the results of the AI, both in relation to the original findings and in relation to the manual count, the results were also convincing. The AI proceeded in the same way to determine the MD as the manual count. The graph shows a high level of agreement (AUC ≈ 0.699), which means that the AI detects the melanocytes well.

Melanocytic nest detection as an additional criterion for LM:

When using individual observer nest detection (GT-N) as the ground truth to evaluate the performance of AI-based nest detection (AI-N), the observer detected melanocytic nests in 25 out of 86 slides (29.07%) in the training set. In comparison, the AI-detected melanocytic nests in 33 out of 86 slides (38.37%). The AI demonstrated higher specificity (87.80%) but lower sensitivity (62.22%), indicating a conservative approach to nest identification that increased the likelihood of missing true positives. The accuracy was 74.42%.

In the next step, we compared the combined AI-MD and AI nest detection (AI-MD-N) with GT-OR and assessed whether this combination improved the classification. The results (sensitivity 88.64%, specificity 64.29%, and accuracy 76.74%) showed no significant improvement in diagnostic accuracy compared to using MD alone.

### 3.2. Test Set

The test set of 177 slides included 15 (8.47%) sample biopsies, 10 (5.65%) were from the centre of a lesion, and 152 (85.88%) from resection margins. No specimens were cut in lamellae. For detailed information, see the supplementary data (Table S2).

Comparison of original findings, AI-assisted melanocyte detection, and manual counts with each other:

Using the same procedure as for the training set, we also determined sensitivity, specificity, and accuracy for the test set, as shown in Table 2.

**Figure 5:**
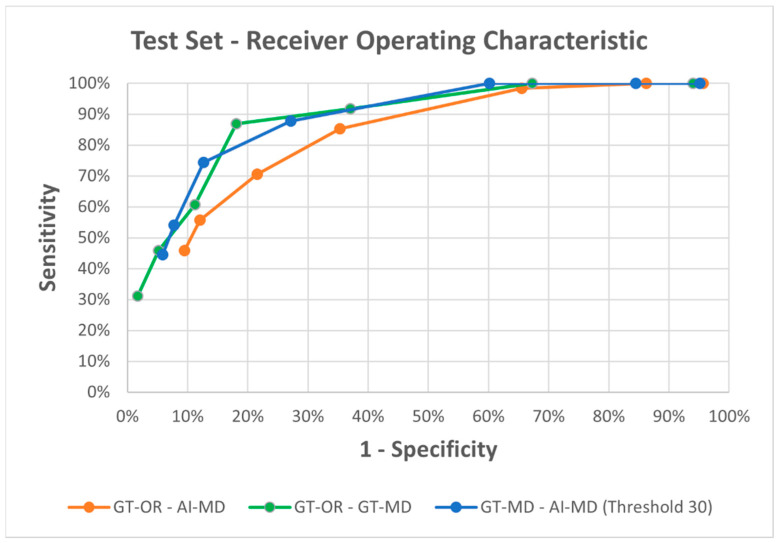
Receiver operator characteristic curve based on Sensitivity (*y*-axis) and 1-Specificity (*x*-axis) comparing AI-generated data based on melanocyte density, ground truth data, and manual cell count-based data in the test set. GT-OR = Ground truth original findings, AI-MD = Artificial intelligence detected melanocyte density; GT-MD = Ground truth melanocyte density.

We again generated ROC curves, which are depicted in Figure 5. We also calculated the AUC of both graphs as above.

AUC (GT-OR vs. GT-MD) ≈ 0.822

AUC (GT-OR vs. AI-MD) ≈ 0.757

AUC (GT-MD (based on 30 melanocytes per 0.5 mm) vs. AI-MD) ≈ 0.818

Here, the AI showed a very high and therefore significantly higher agreement with manual counting than in the training set. These results were very encouraging and demonstrated good melanocyte detection. The results also significantly improved in comparison to the original findings.

Detection of melanocytic nests as an additional criterion for LM:

Nests were manually detected in 35 slides (19.77%) compared to 64 slides (36.16%) detected by AI. The AI nest detection (GT-AI) compared to the ground truth nest (GT-N) now showed a higher sensitivity (88.57%) compared to the training set, but at the expense of specificity (76.06%). The accuracy was 78.53%.

Adding AI-based nest detection to AI-based MD analysis alone did not improve accuracy (67.80%). Sensitivity and Specificity were at 93.44% and 54.31%, respectively.

**Figure 6:**
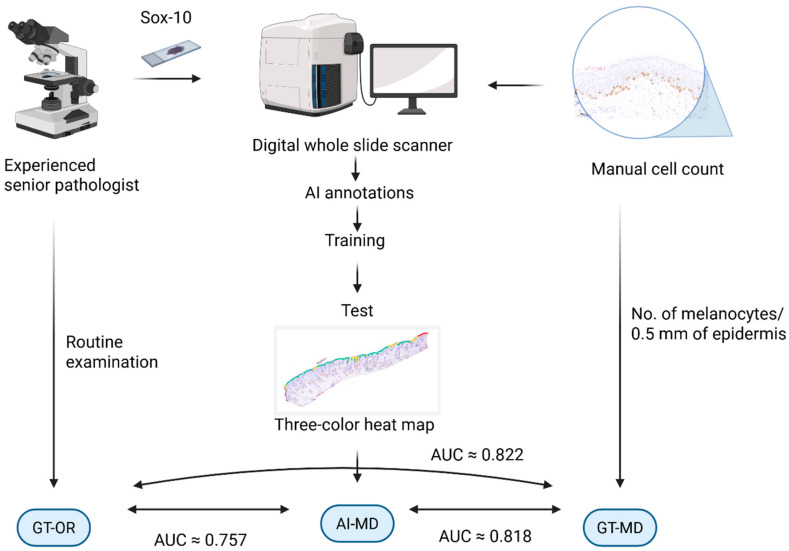
Graphical summary of approach and results of the study. Created in BioRender. Mitteldorf, C. (2025) https://BioRender.com/bbr0p10.

To summarise, Figure 6 provides a simplified illustration of the procedure and the results in a quick overview.

## 4. Discussion

Our data demonstrated that our AI model can effectively detect LM based on MD using Sox-10 nuclear staining, achieving high sensitivity and specificity. The best performance was seen in direct comparison with manual counting of the GT-MD, suggesting that the AI model successfully mirrored the manual counting of melanocytes and therefore showed a satisfactory detection of melanocytes. An analysis of the ROC curves and the AUC values of GT-OR vs. GT-MD, GT-MD vs. AI-MD, and GT-OR vs. AI-MD showed high integrals, especially in the test set. We concluded that the MD is a good marker for the assessment of resection margins in an LM lesion, but also that the AI determines them well and shows a high degree of agreement with the original findings.

As mentioned above, only one paper addressed a deep learning model for resection margin assessment in LM [42]. In this study, the AI model was trained to detect melanocytes in annotated Sox-10-stained slides (training set: *n* = 65), and then the trained AI model was applied to detect melanocytes in H&E (test set: *n* = 58) [42]. One advantage mentioned by the authors was the elimination of expensive and time-consuming immunohistochemical staining [42]. Today, the use of immunohistochemical stains for the diagnosis of LM is considered a recommended standard of care; in particular, nuclear stains show clear advantages over H&E [5,47,48,49,50]. Our AI was trained to detect nuclear immunohistochemical signals in Sox-10 and can also be applied to other nuclear markers. The nuclear marker PRAME, for example, provides valuable additional diagnostic information, particularly in the evaluation of surgical margins and in lesions with increased melanocyte density due to concurrent entities [5,50]. Not only do such stains improve diagnostic accuracy, but the absence of immunohistochemical staining may result in minimally invasive tumour components being easily missed. Sox-10 staining, in particular, but also MITF as another nuclear stain, is superior to conventional H&E in detecting LM, resulting in better LM detection and is also associated with generally higher MD and with a reduced interobserver variability compared to H&E [47,48,49]. Interestingly, Jackson et al. have developed a machine learning algorithm that mimics immunohistochemical staining (based on Sox-10) [51]. Further development of this AI would obviously be very helpful, as it would eliminate the need for expensive and time-consuming staining of often limited tissue [51].

Siarov et al. [42] reported substantial interobserver agreement (κ = 0.62) when evaluations were based on H&E-stained specimens alone, without the assistance of AI. The integration of AI led to a moderate improvement in agreement (κ = 0.69); however, this remained within the same categorical level of “substantial agreement” and thus did not represent a qualitative leap according to established classification systems. From a clinical perspective, a higher level of interobserver agreement would be highly desirable to improve diagnostic reliability and consistency. They showed that the evaluation by junior dermatopathologists could be improved by using AI, which can be very useful in clinical routine [42]. The improvement in the workflow of experienced dermatopathologists was not statistically significant [42]. Here, the AI we developed offers the crucial advantage of saving time by recognising the epidermis and visualising a heat map. Both inexperienced and experienced dermatopathologists can thus benefit.

Siarov et al. achieved a high AUC value (0.84) in their AI model compared to the manually determined MD [42]. Nonetheless, our results can also compete very well with an AUC of 0.818. However, in the case of the study just mentioned, the results are based on a cut-off of ≥ 25 melanocytes per 0.5 mm of epidermis as a high risk of recurrence, and anything below that as a low risk [42]. Here, they refer to the results of a study by Gorman et al. [46]. The cut-off value may be set too low, as it was derived from evaluations based on H&E-stained specimens [42,46,47]. In our work, we determined the most appropriate cut-off value for our training collective ourselves and ended up with 30 melanocytes per 0.5 mm, which is consistent with the average increased MD for Sox-10 in LM compared to H&E-stained slides [47]. Additionally, a borderline zone can be defined, which in our setting was 25–29 melanocytes/0.5 mm of epidermis. All parameters can be customised. This gave us the advantage of being able to generate a three-colour heat map that was automatically generated over the entire slide by detection and automated length measurement of the epidermis and of the epithelium of the hair follicle infundibulum. In contrast, the previously published deep learning model required manual measurement and selection of the epidermis [42]. Manually measuring the epidermis and drawing the area of interest results in a time-consuming process. In addition, areas not manually marked could easily be missed. Moreover, high melanocyte densities can also occur in short segments of the epidermis measuring less than 0.5 mm in length. These areas may not be adequately assessed by conventional AI models, which rely on manual measurements. However, our automated epidermis detection and measurement algorithm was specifically trained to accurately evaluate melanocyte densities in both short and long epidermal segments (see also Figure 2). Our AI model can therefore save a lot of time in routine examinations and may reduce the high interobserver variability in the future. Moreover, discrete affected areas, especially in the edges and on fragmented slides, which can be easily overlooked, were highlighted by the AI. In addition, a fatigue-related variability in diagnostic accuracy could be reduced. In turn, the model can focus directly on severely affected areas. Finally, the AI-generated results could be reviewed and approved by the (dermato) pathologist.

Requa et al. were able to train an AI model that not only diagnosed five main types of cutaneous tumours, but also classified them into subtypes and provided information on localisation and margin status [24]. It shows a very high sensitivity for all entities [24]. However, these promising results were obtained only with H&E staining [24]. A transfer to immunohistochemical stains would be desirable. Nevertheless, the large number of samples included in the study remains impressive [24].

MD is only one criterion for evaluating LM, and other criteria such as adnexal extension and pagetoid spread remain important in the assessment [52]. Especially, the involvement of melanocytes spreading into the follicular-adnexal areas is an important malignancy criterion. That was the reason why the AI tool is also able to recognise the infundibula of the hair follicles. Nest formations can also be an additional criterion in LM detection [10]. Kucharski et al. have previously developed a deep learning tool that detects melanocyte nests in H&E-stained sections of various melanocytic entities using autoencoders [53]. While the AI we used demonstrated acceptable nest detection capabilities, this feature did not significantly improve diagnostic performance in the training set. The addition of nests only slightly improved sensitivity in the test set and reduced overall accuracy. The high rate of AI-detected false positives may increase pathologist review time without adding substantial diagnostic value. From a practical perspective, we believe that MD is the most reliable and quantifiable parameter for the detection of LM, with realistic potential for implementation in routine clinical practice. In addition, in most cases, a nest leads to an increased MD, which in turn is recognised by the AI model and is therefore included in the evaluation anyway. Therefore, even if we are aware that focusing on MD alone can lead to misinterpretations, and that other malignancy criteria are just as important. Nevertheless, MD is an extremely important criterion that can also be quantified. This can quickly provide an overview for the examiner.

The following factors contributed to a more challenging assessment of the specimens by the AI. Dirt on the slides and applied colour markings initially led to false positive results, but could usually be eliminated by retraining the AI. More problematic was the collision with additional lesions (e.g., lentigo solaris, seborrheic keratosis, and (pigmented) actinic keratosis) that also have an elevated MD. A combination with a second model that detects these additional tumours would be ideal. Other problems were tangentially hit sections and poor staining quality, but this is also a challenge in conventional diagnostics. Despite these limitations, one major strength of the AI model is its ability to highlight problematic areas for further review. This ensures that ambiguous regions are not missed, even in difficult or fragmented specimens.

Previous work has shown an AI that has also been trained to recognise the epidermis, melanocytes, and keratinocytes, and can distinguish between melanotic nevus, melanoma, and normal skin with a high accuracy of about 90% [54]. Our work has the advantage that the examiner immediately receives a visual aid from the traffic light system and can thus quickly confirm the diagnosis provided by the AI.

Of course, there is a risk that the examiner will be influenced by the AI tool. However, the benefits of efficiency, standardisation, and increased sensitivity—especially for subtle or peripheral findings—may outweigh this risk. A heat overview allows the examiner to focus on the relevant areas at first glance, so that they can be assessed first, making the evaluation more efficient. In addition, the MD is a quantitative, tangible value that standardises work processes [7]. The cut-off value can be adjusted as needed. In our case, we achieved the best results with a cut-off of 30 melanocytes per 0.5 mm. This is certainly not easily transferable, but can be a rough guide. A limitation of the study is a small dataset from a single centre. As a result, given its exploratory nature and limited cohort size, we did not perform a formal confidence interval analysis; the reported sensitivities, specificities, and accuracies should therefore be interpreted as descriptive preliminary estimates.

A follow-up project is planned, in which a larger number of samples from different laboratories will be analysed to validate our results.

## 5. Conclusions

Our AI model effectively detects LM by identifying MD using Sox-10 nuclear staining, achieving high sensitivity and specificity. It closely mirrors the manual counting of melanocytes, with promising results compared to human assessment. Our model outperformed previously published deep learning models by automatically detecting the epidermis, generating a colour-coded heat map of MD, and eliminating the need for manual measurement. This approach saves time and reduces interobserver variability. Although further training is necessary, our AI model holds significant potential to improve diagnostic efficiency, reduce observer variability, and aid in routine clinical practice by streamlining LM evaluation.

This article is a revised and expanded version of a research poster entitled ‘Schnittrandkontrolle der Lentigo maligna mit Hilfe künstlicher Intelligenz’, which was presented at the Annual Meeting of the German Society of Dermatopathology (Göttingen, Germany: 14th to 16th June 2024) [55].

## Figures and Tables

**Table 1:** Sensitivity, specificity, and accuracy of the comparison between the AI-generated data based on melanocyte density with the ground truth data and the manual cell count-generated data, and between the ground truth data with the manual cell count-based data in the training set. GT-MD = Manual cell count ground truth data, AI-MD = Data generated by the AI; GT-OR = Ground truth data of the original findings.

	GT-MD ↔ AI-MD	GT-OR ↔ AI-MD	GT-OR ↔ GT-MD
Sensitivity (%)	88.89	85.71	95.24
Specificity (%)	75.61	68.18	88.64
Accuracy (%)	82.56	76.74	91.86

**Table 2:** Sensitivity, specificity, and accuracy of the comparison between the AI-generated data based on melanocyte density with the ground truth data and the manual cell count-generated data, and between the ground truth data with the manual cell count-based data in the test set. GT-MD = Manual cell count ground truth data, AI-MD = Data generated by the AI; GT-OR = Ground truth data of the original findings.

	GT-MD ↔ AI-MD	GT-OR ↔ AI-MD	GT-OR ↔ GT-MD
Sensitivity (%)	87.84	85.25	86.89
Specificity (%)	72.82	64.66	81.90
Accuracy (%)	79.10	71.75	83.62

## Data Availability

The raw data supporting the conclusions of this article will be made available by the authors on request.

## Supplementary Materials

The following supporting information can be downloaded at: https://www.mdpi.com/article/10.3390/dermatopathology13010001/s1, Table S1: This shows the percentage of each individual excision source that was classified as positive or negative in the training set; Table S2: This shows the percentage of each individual excision source that was classified as positive or negative in the test set.
