# Supplementary material for: Artificial Intelligence for Lentigo Maligna: Automated Margin Assessment via Sox-10-Based Melanocyte Density Mapping
Source: Dermatopathology (Basel). 2025 Dec 19;13(1):1. doi: 10.3390/dermatopathology13010001 (PMC12821717; doi:10.3390/dermatopathology13010001)
Supplement: Supplementary file 1 [file dermatopathology-13-00001-s001.zip › dermatopathology-3956303-supplementary.pdf]

Localisation	Total number	Percentage of all training sections	Rated as positive		Rated as negative	
			Number of cases	percentage of individual localisation (%)	Number of cases	percentage of individual localisation (%)
SB	5	5.81	5	100.0	0	0.0
C	12	13.95	11	91.67	1	8.33
RM	66	76.74	23	34.85	43	65.15
S	3	3.49	3	100.0	0	0.0

Table S1: This shows the percentage of each individual excision source that was classified as positive or negative in the **training set**. SB=sample biopsy, C=centre, RM= resection margin, S= spindle

Localisation	Total number	Percentage of all training sections	Rated as positive		Rated as negative	
			Number of cases	percentage of individual localisation (%)	Number of cases	percentage of individual localisation (%)
SB	15	8.47	3	20.0	12	80.0
C	10	5.65	8	80.0	2	20.0
RM	152	85.88	52	34.21	100	65.79

Table S2: This shows the percentage of each individual excision source that was classified as positive or negative in the **test set**. SB=sample biopsy, C=centre, RM= resection margin
